# Prospective validation of the 4C prognostic models for adults hospitalised with COVID-19 using the ISARIC WHO Clinical Characterisation Protocol

**DOI:** 10.1136/thoraxjnl-2021-217629

**Published:** 2021-11-21

**Authors:** Stephen R Knight, Rishi K Gupta, Antonia Ho, Riinu Pius, Iain Buchan, Gail Carson, Thomas M Drake, Jake Dunning, Cameron J Fairfield, Carrol Gamble, Christopher A Green, Sophie Halpin, Hayley E Hardwick, Karl A Holden, Peter W Horby, Clare Jackson, Kenneth A Mclean, Laura Merson, Jonathan S Nguyen-Van-Tam, Lisa Norman, Piero L Olliaro, Mark G Pritchard, Clark D Russell, Catherine A Shaw, Aziz Sheikh, Tom Solomon, Cathie Sudlow, Olivia V Swann, Lance C W Turtle, Peter J M Openshaw, J Kenneth Baillie, Annemarie Docherty, Malcolm G Semple, Mahdad Noursadeghi, Ewen M Harrison, ISARIC Coronavirus Clinical Characterisation Consortium (ISARIC4C) Investigators, J Kenneth Baillie

**Affiliations:** 1 Centre for Medical Informatics, The University of Edinburgh, Usher Institute of Population Health Sciences and Informatics, Edinburgh, UK; 2 University College London Institute for Global Health, London, UK; 3 Medical Research Council University of Glasgow Centre for Virus Research, Glasgow, UK; 4 Manchester Academic Health Science Centre, Manchester, UK; 5 Department of Public Health and Policy, University of Liverpool, Liverpool, UK; 6 Nuffield Department of Clinical Medicine, ISARIC Global Support Centre, Centre for Tropical Medicine and Global Health, University of Oxford, Oxford, UK; 7 Public Health England National Infection Service, Salisbury, UK; 8 National Heart and Lung Institute, Imperial College London, London, UK; 9 Liverpool Clinical Trials Centre, University of Liverpool, Liverpool, UK; 10 Institute of Microbiology and Infection, University of Birmingham, Birmingham, UK; 11 NIHR Health Protection Research Unit, Institute of Infection, Veterinary and Ecological Sciences, Faculty of Health and Life Sciences, University of Liverpool, Liverpool, UK; 12 Division of Epidemiology and Public Health, University of Nottingham, Nottingham, UK; 13 Nuffield Department of Medicine, Centre for Tropical Medicine and Global Health, University of Oxford, Oxford, UK; 14 Queen’s Medical Research Institute, University of Edinburgh, Edinburgh, UK; 15 Usher Institute of Population Health Sciences and Informatics, University of Edinburgh, Edinburgh, UK; 16 Health Data Research UK, London, UK; 17 Department of Child Life and Health, University of Edinburgh, Edinburgh, UK; 18 Clinical Infection, Microbiology and Immunology, University of Liverpool Faculty of Health and Life Sciences, Liverpool, UK; 19 Liverpool University Hospitals Foundation Trust, Member of Liverpool Health Partners, Liverpool, UK; 20 Respiratory Department, Imperial College, London, UK; 21 Genetics and Genomics, Roslin Institute, University of Edinburgh, Edinburgh, UK; 22 Respiratory Medicine, Alder Hey Children's Hospital, University of Liverpool, Liverpool, UK; 23 Division of Infection and Immunity, University College London, London, UK

**Keywords:** COVID-19

## Abstract

**Purpose:**

To prospectively validate two risk scores to predict mortality (4C Mortality) and in-hospital deterioration (4C Deterioration) among adults hospitalised with COVID-19.

**Methods:**

Prospective observational cohort study of adults (age ≥18 years) with confirmed or highly suspected COVID-19 recruited into the International Severe Acute Respiratory and emerging Infections Consortium (ISARIC) WHO Clinical Characterisation Protocol UK (CCP-UK) study in 306 hospitals across England, Scotland and Wales. Patients were recruited between 27 August 2020 and 17 February 2021, with at least 4 weeks follow-up before final data extraction. The main outcome measures were discrimination and calibration of models for in-hospital deterioration (defined as any requirement of ventilatory support or critical care, or death) and mortality, incorporating predefined subgroups.

**Results:**

76 588 participants were included, of whom 27 352 (37.4%) deteriorated and 12 581 (17.4%) died. Both the 4C Mortality (0.78 (0.77 to 0.78)) and 4C Deterioration scores (pooled C-statistic 0.76 (95% CI 0.75 to 0.77)) demonstrated consistent discrimination across all nine National Health Service regions, with similar performance metrics to the original validation cohorts. Calibration remained stable (4C Mortality: pooled slope 1.09, pooled calibration-in-the-large 0.12; 4C Deterioration: 1.00, –0.04), with no need for temporal recalibration during the second UK pandemic wave of hospital admissions.

**Conclusion:**

Both 4C risk stratification models demonstrate consistent performance to predict clinical deterioration and mortality in a large prospective second wave validation cohort of UK patients. Despite recent advances in the treatment and management of adults hospitalised with COVID-19, both scores can continue to inform clinical decision making.

**Trial registration number:**

ISRCTN66726260.

**Key messages**

**What is the key question?**

- The clinical characteristics and management of hospitalised patients with confirmed or highly suspected COVID-19 have changed over time; ongoing prospective validation of risk prediction scores is therefore required.

**What is the bottom line?**

- Both 4C prediction scores performed well in a large prospective UK cohort, with stable validation metrics across National Health Service regions and ethnicity, despite reduced overall deterioration and mortality risk compared with original derivation and validation cohorts.

**Why read on?**

- This is the first large prospective revalidation of these risk stratification tools, which demonstrated stable performance in over 75 000 hospitalised UK patients.

## Introduction

Disease resulting from infection with SARS-CoV-2 has a high mortality rate with deaths predominantly caused by respiratory failure.^1^ We previously reported two prognostic scores for in-hospital mortality^2^ and deterioration^3^ from the International Severe Acute Respiratory and Emerging Infections Consortium Coronavirus Clinical Characterisation Consortium (ISARIC4C) study derived and validated in large UK cohorts during the first pandemic wave.

As hospitals around the world faced a continued influx of patients with COVID-19, these easy-to-use risk stratification tools have facilitated early identification of patients infected with SARS-CoV-2 who are at the highest risk of deterioration and death to guide management and optimise resource allocation.^4^ Both scores use readily available clinical and biochemical parameters at the time of assessment, with predicted risk used to guide antiviral treatment across the UK.^5^ Furthermore, independent external validation has demonstrated consistent performance worldwide.^6–10^

Management and treatment of patients admitted or diagnosed in hospital with COVID-19 have changed markedly over the past year, notably with the introduction of corticosteroids for people requiring supplemental oxygen or ventilatory support.^11^ Ongoing prospective validation is therefore necessary to ensure adequate performance and inform the need for temporal recalibration.^12^

In this article, we extend our previous work by prospectively validating these scores in a large study cohort using the wide geographical coverage of the ISARIC4C study cohort in England, Wales, and Scotland, among adults recruited during the second pandemic wave.

## Methods

### Study population and data collection

The International Severe Acute Respiratory and emerging Infections Consortium (ISARIC)–WHO Clinical Characterisation Protocol UK (CCP-UK) study is an ongoing prospective multicentre cohort study being conducted by ISARIC4C in 306 hospitals across England, Scotland and Wales (National Institute for Health Research (NIHR) Clinical Research Network Central Portfolio Management System ID 14152). The study was part of a suite of ‘sleeping’ protocols established and approved in the UK prior to the pandemic and was activated on 17 January 2020. CCP-UK is a version of a standardised open-source international research protocol (the ISARIC/WHO CCP) created in 2012, which has been employed worldwide for harmonised observational studies of COVID-19.^13–15^

The CCP-UK protocol and further study details are available online.^16^ In this analysis, we included consecutive adults (aged ≥18 years) who had highly suspected or PCR-confirmed COVID-19. As before, we included patients with suspected COVID-19 in the analysis because both models were intended for use in participants at the point of initial evaluation for COVID-19, when virological confirmation might not be available. We also included nosocomial COVID-19 acquisition to test the hypothesis that acquisition of infection in hospital might be associated with differential risk. Community-acquired infection was defined as symptom onset or first positive SARS-CoV-2 PCR result within 7 days from admission; participants who did not meet these criteria and had either symptom onset or first positive SARS-CoV-2 PCR result more than 7 days from admission were classified as nosocomial cases.^17^ Among nosocomial cases, patients who met the deterioration outcome before the onset of COVID-19 were excluded. Northern Ireland was excluded from model validation due to the small numbers of patients recruited.

For both scores, we included eligible hospitalised participants with confirmed or highly suspected COVID-19 between 27 August 2020 and 17 February 2021. All patients had at least four weeks follow-up to reduce selection bias (final data extraction date: 23 April 2021). Patients included within original derivation or validation cohorts for either score were excluded from this analysis. Participants who had ongoing hospital care at the end of follow-up (the point at which a final outcome was recorded in the case record form) were classified as not meeting the endpoint because the risk of deterioration declines with time since admission.^3^ Hospitals that recruited participants admitted to intensive care unit (ICU) exclusively were not included since these participants had already met the 4C Deterioration outcome by definition. Both scores are summarised in table 1, with additional information on how coefficients were transformed into points systems contained in online supplemental appendix 1.

10.1136/thoraxjnl-2021-217629.supp1 Supplementary data

**Table 1:** Model parameters for 4C Mortality and 4C Deterioration prognostic model

4C Mortality score			4C Deterioration	
Variable			Characteristic	Log(OR)
Age (years)	<50	–	Intercept	4.033
	50–59	+2		
	60–69	+4	Age (years)	0.0159
	70–79	+6	Age (spline 1)	−0.0129
	≥80	+7	Age (spline 2)	0.1265
Sex	Female	–	Sex	
	Male	+1	Female	–
			Male	0.2439
Number of comorbidities	0	–	Nosocomial	
	1	+1	No	–
	≥2	+2	Yes	0.2439
Respiratory rate (breaths/minute)	<20	–	Radiographic infiltrates	
	20–29	+1	No	–
	≥30	+2	Yes	0.3252
Peripheral oxygen saturation on room air (%)	≥92	–	Respiratory rate (breaths/minute)	−0.0145
	<92	+2	Respiratory rate (spline 1)	0.5992
			Respiratory rate (spline 2)	−1.078
Glasgow Coma Scale score	15	–		
	<15	+2		
Urea (mmol/L)	<7	–	Peripheral oxygen saturation (SpO_2_)	−0.07078
	7–14	+1	SpO_2_ (spline 1)	−0.0248
	>14	+3	SpO_2_ (spline 2)	1.024
C reactive protein (mg/L)	<50	–	Room air or oxygen	
	50–99	+1	Room air	–
	≥100	+2	Oxygen therapy	0.7450
			Glasgow Coma Scale score	
			15	–
			<15	0.6028
			Urea (mmol/L)	0.0508
			Urea (spline 1)	0.4446
			Urea (spline 2)	−1.035
			C reactive protein (mg/L)	0.0097
			C reactive protein (spline 1)	−0.0395
			C reactive protein (spline 2)	0.0588
			Lymphocyte count (x10^9^/L)	−0.4564
			Lymphocytes (spline 1)	0.7309
			Lymphocytes (spline 2)	−0.8113

Restricted cubic spline knot positions are:

Age = 38.5, 67.7, 81.1, 92.9;

Respiratory rate=16, 19, 24, 37;

SpO_2_=84, 94, 96, 100;

Urea = 2.9, 5.7, 9.2, 25.5;

C reactive protein=5, 45, 113, 297;

Lymphocytes = 0.3, 0.7, 1.1, 2.4.

The study is reported in accordance with Transparent Reporting of a multivariable prediction model for Individual Prognosis Or Diagnosis (TRIPOD) guidance.^18^ Demographic, clinical and outcome data were collected by research nurses and medical student volunteers using a publicly available standardised case record form and uploaded to a Research Electronic Data Capture Database (REDCap, Vanderbilt University, US, hosted by University of Oxford, UK). Co-morbidities were defined by a modified Charlson comorbidity index and obesity was clinician-defined.^16^ Consent was not required for the use of routinely collected clinical data from medical records. The Control of Patient Information notice 2020 for urgent public health research makes provision for this in England and Wales. A waiver for consent was obtained from the Public Benefit and Privacy Panel for Health and Social Care in Scotland.

### Outcomes

For the 4C Deterioration model, we used a composite primary outcome comprising any of the following during hospital admission and were equally weighted: initiation of ventilatory support (non-invasive ventilation, invasive mechanical ventilation or extracorporeal membrane oxygenation); admission to a high-dependency unit or ICU; or death (all-cause), as reported previously.^3^

### Model validation

All validation analyses were conducted as described in our previous publication.^3^ Briefly, we assessed model discrimination (how well predictions differentiated participants who experienced the outcomes from those who did not, quantified as the C-statistic) and calibration (agreement between predicted and observed risk, assessed using calibration slopes, calibration-in-the-large and calibration plots).^19 20^ To assess calibration for the 4C Mortality score, we transformed points scores to the probability scale using the observed mortality proportion for each distinct total point score in the original reported validation cohort.^2^

We used multiple imputation with chained equations to account for missing data using the mice package in R,^21^ as previously described.^3^ We included all predictors (including restricted cubic spline transformations) and the outcome in the imputation models. Analyses were done in each of the 10 multiply imputed datasets and pooled using Rubin’s rules.^22^ Our primary analyses were performed stratified by National Health Service (NHS) region in order to examine for evidence of between-region heterogeneity in model performance. We visualised C-statistics, calibration-in-the-large and slope estimates across regions in forest plots and calculated pooled estimates using random effects meta-analysis, as previously recommended.^23^

Decision curve analysis allows assessment of clinical utility by quantifying the trade-off between correctly identifying true positives and incorrectly identifying false positives weighted according to the threshold probability.^24^ The threshold probability represents the risk cut-off above which any given treatment or intervention might be considered and reflects the perceived risk:benefit ratio for the intervention. Decision curve analysis was used to quantify the net benefit of implementing the model in clinical practice^24^ compared with the following: a treat-all approach; a treat-none approach. All decision curves were smoothed by locally weighted smoothing (LOESS) from stacked multiply imputed datasets.

A sensitivity analysis was also performed, with stratification of the validation cohort by ethnic group and month of admission, in view of previously reported differences in COVID-19 outcomes by ethnicity and over time.^25 26^ All analyses were done in R (V.3.6.3).

### Patient and public involvement

This was an urgent public health research study in response to a Public Health Emergency of International Concern. Patients or the public were not involved in the design, conduct or reporting of this rapid response research.

### Role of the funding source

The funder of the study had no role in study design, data collection, data analysis, data interpretation or writing of the report. All authors had full access to all the data in the study and had final responsibility for the decision to submit for publication.

## Results

**Figure 1:**
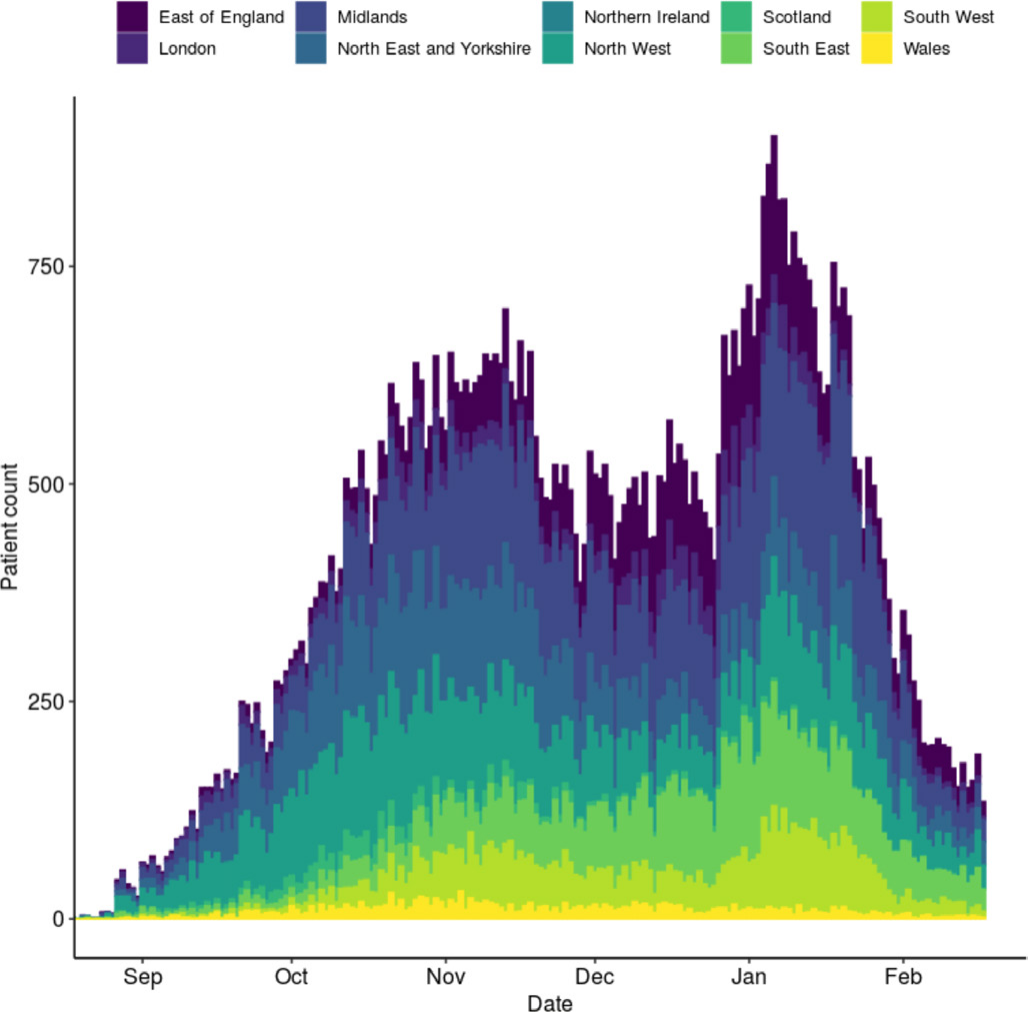
Histogram showing dates of hospital admission or first COVID-19 assessment for adults included in this analysis, stratified by National Health Service region.

Between 27 August 2020 and 17 February 2021, 76 588 eligible adults were recruited to the ISARIC4C study and included in the current analysis, of whom 69 260 (90.4%) were known to have PCR-confirmed COVID-19. Baseline demographic, physiological and laboratory characteristics are shown stratified by outcome (table 2). The median age of patients in the cohort was 72 years (IQR 57–83); 35 231 (46.0%) were female and 52 704 (72.6%) had at least one comorbidity. The temporal distribution of participant admissions, stratified by NHS region, is shown in figure 1. For patients with nosocomial infections, the median time from admission to recruitment was 15 days (IQR 10–29). A summary of missingness for predictor variables included in both scores is shown in online supplemental appendix 2.

**Table 2:** Baseline characteristics, stratified by first chronological deterioration category through which they met the composite 4C Deterioration primary outcome (HDU admission, ICU admission or ventilatory support, or death)

Characteristic	Overall, N=76 588*	Ventilatory support or HDU/ICU, N=14 771*	Died, N=12 581*	No deterioration, N=45 726*	(Missing), N=3510*
Age (years)	72 (57–83)	65 (54–75)	84 (77–89)	70 (54–82)	75 (60–84)
Missing	27 (<0.1)	4 (<0.1)	5 (<0.1)	7 (<0.1)	11 (<0.1)
Sex					
Female	35 231 (46)	5307 (36)	5533 (44)	22 717 (50)	1674 (48)
Male	41 250 (54)	9447 (64)	7028 (56)	22 955 (50)	1820 (52)
Missing	107 (0.1)	17 (0.1)	20 (0.2)	54 (0.1)	16 (0.5)
Ethnicity					
White	56 578 (85)	10 063 (78)	10 291 (92)	33 567 (84)	2657 (90)
South Asian	4098 (6.1)	1179 (9.2)	294 (2.6)	2565 (6.4)	60 (2.0)
Black	1536 (2.3)	389 (3.0)	95 (0.9)	1005 (2.5)	47 (1.6)
East Asian	337 (0.5)	105 (0.8)	32 (0.3)	188 (0.5)	12 (0.4)
Other	4257 (6.4)	1108 (8.6)	423 (3.8)	2565 (6.4)	161 (5.5)
Missing	9782 (13)	1927 (13)	1446 (11)	5836 (13)	573 (16)
SARS-CoV-2 PCR positive	69 260 (99)	13 226 (99)	11 642 (99)	41 935 (99)	2457 (99)
Missing	6841 (8.9)	1432 (9.7)	873 (6.9)	3502 (7.7)	1034 (29)
Number of comorbidities	1 (0–2)	1 (0–2)	2 (1–3)	1 (0–2)	1 (0–2)
One or more comorbidities	52 704 (73)	10 118 (71)	10 865 (91)	30 102 (68)	1619 (46)
Missing	3985 (5.2)	478 (3.2)	621 (4.9)	1674 (3.7)	1212 (35)
Nosocomial infection	9603 (13)	553 (3.8)	2489 (20)	6088 (14)	473 (16)
Missing	1973 (2.6)	132 (0.9)	329 (2.6)	1027 (2.2)	485 (14)
Radiographic infiltrates	26 079 (64)	8320 (81)	3563 (59)	13 485 (58)	711 (54)
Missing	35 735 (46)	4533 (31)	6569 (52)	22 450 (49)	2183 (62)
Respiratory rate (per min)	20 (18–24)	24 (20–30)	20 (18–24)	20 (18–23)	20 (18–23)
Missing	6535 (8.5)	760 (5.1)	962 (7.6)	3431	1382 (39)
SpO_2_ (%)	96 (93–97)	94 (89–96)	96 (93–97)	96 (94–98)	96 (94–97)
SpO_2_ <94%	18 897 (27)	6672 (48)	3324 (24)	8373 (74)	528 (3.9)
Missing	6401 (8.4)	763 (5.1)	964 (7.7)	3405 (7.4)	1269 (36)
Room air or oxygen					
Room air	47 061 (68)	5885 (42)	7810 (68)	31 831 (76)	1535 (70)
Oxygen	22 384 (32)	8018 (58)	3655 (32)	10 038 (24)	673 (30)
Missing	7143 (9.3)	868 (5.9)	1116 (8.9)	3857 (8.4)	1302 (37)
Glasgow Coma Scale	15 (15–15)	15 (15–15)	15 (15–15)	15 (15–15)	15 (15–15)
Missing	6332 (8.2)	873 (5.9)	1189 (9.5)	2780 (6.1)	1490 (42)
Lymphocytes (x10ˆ9 /L)	0.90 (0.60–1.30)	0.80 (0.56–1.10)	0.80 (0.50–1.18)	0.98 (0.70–1.40)	0.90 (0.60–1.27)
Missing	16 087 (21)	1448 (11)	3073 (24)	10 778 (23.6)	788 (22)
Urea (mmol/L)	6.6 (4.6–9.9)	6.9 (4.9–10.3)	9.7 (6.6–15.1)	5.9 (4.3–8.6)	6.5 (4.6–9.7)
Missing	20 552 (27)	2403 (16)	3701 (29)	13 405 (29)	1043
C reactive protein (mg/L)	69 (27–133)	109 (57–181)	76 (34–142)	55 (20–110)	57 (22–114)
Missing	21 543 (28)	2262 (15)	3863 (31)	14 320 (31)	1098
NHS region					
East of England	9507 (12)	1446 (9.8)	1819 (14)	5132 (11)	1110 (32)
London	3886 (5.1)	1272 (8.6)	306 (2.4)	2184 (4.8)	124 (3.5)
Midlands	17 988 (23)	3043 (21)	3100 (25)	11 333 (25)	512 (15)
North East and Yorkshire	12 081 (16)	2465 (17)	1845 (15)	7520 (16)	251 (7.2)
Northern Ireland	31 (<0.1)	17 (0.1)	1 (<0.1)	13 (<0.1)	0 (0)
North West	13 731 (18)	2847 (19)	2321 (18)	8120 (18)	443 (13)
Scotland	1558 (2.0)	340 (2.3)	205 (1.6)	916 (2.0)	97 (2.8)
South East	9530 (12)	1903 (13)	1619 (13)	5672 (12)	336 (9.6)
South West	6485 (8.5)	1079 (7.3)	991 (7.9)	3788 (8.3)	627 (18)
Wales	1791 (2.3)	359 (2.4)	374 (3.0)	1048 (2.3)	10 (0.3)
Oxygen received	49 894 (69)	14 277 (98)	9777 (81)	24 859 (56)	981 (79)
Missing	4249 (5.6)	176 (1.2)	546 (4.3)	1264 (2.8)	2263
Systemic steroids received	38 417 (54)	11 769 (84)	6765 (57)	19 617 (44)	266 (47)
Missing	5944 (7.8)	684 (4.6)	686 (5.5)	1636 (3.6)	2938
Deterioration	27 352 (37)	14 771 (100)	12 581 (100)	0 (0)	0 (0)
Missing	3510 (4.6)	0 (0.0)	0 (0.0)	0 (0.0)	3510
Mortality	18 211 (25)	5630 (40)	12 581 (100)	0 (0)	0 (0)
Missing	4107 (5.4)	597 (4.0)	0 (0.0)	0 (0.0)	3510
Length of stay (days)	10 (5–19)	12 (7–21)	13 (6–22)	8 (4–17)	7 (1–23)
Missing	6888 (9.0)	1170 (7.9)	358 (2.8)	1867 (4.1)	3493

Data are median (IQR) or n (%), calculated from non-missing data. Participants are shown by the first chronological deterioration category through which they met the composite primary outcome (HDU or ICU admission, ventilatory support or death).

*Statistics presented: median (IQR); n (%).

HDU, high-dependency unit; ICU, intensive care unit; NHS, National Health Service.;

For the deterioration outcome, 73 078 (95.4%) participants had an outcome available, and in-hospital clinical deterioration occurred in 27 352 (37.4%), with a median time to deterioration of 5 days (IQR 1–12). For mortality, 72 481 (94.6%) participants had an outcome available, with in-hospital death occurring among 12 581 (17.4%). The median time to death was 11 days (IQR 6–18).

**Figure 2:**
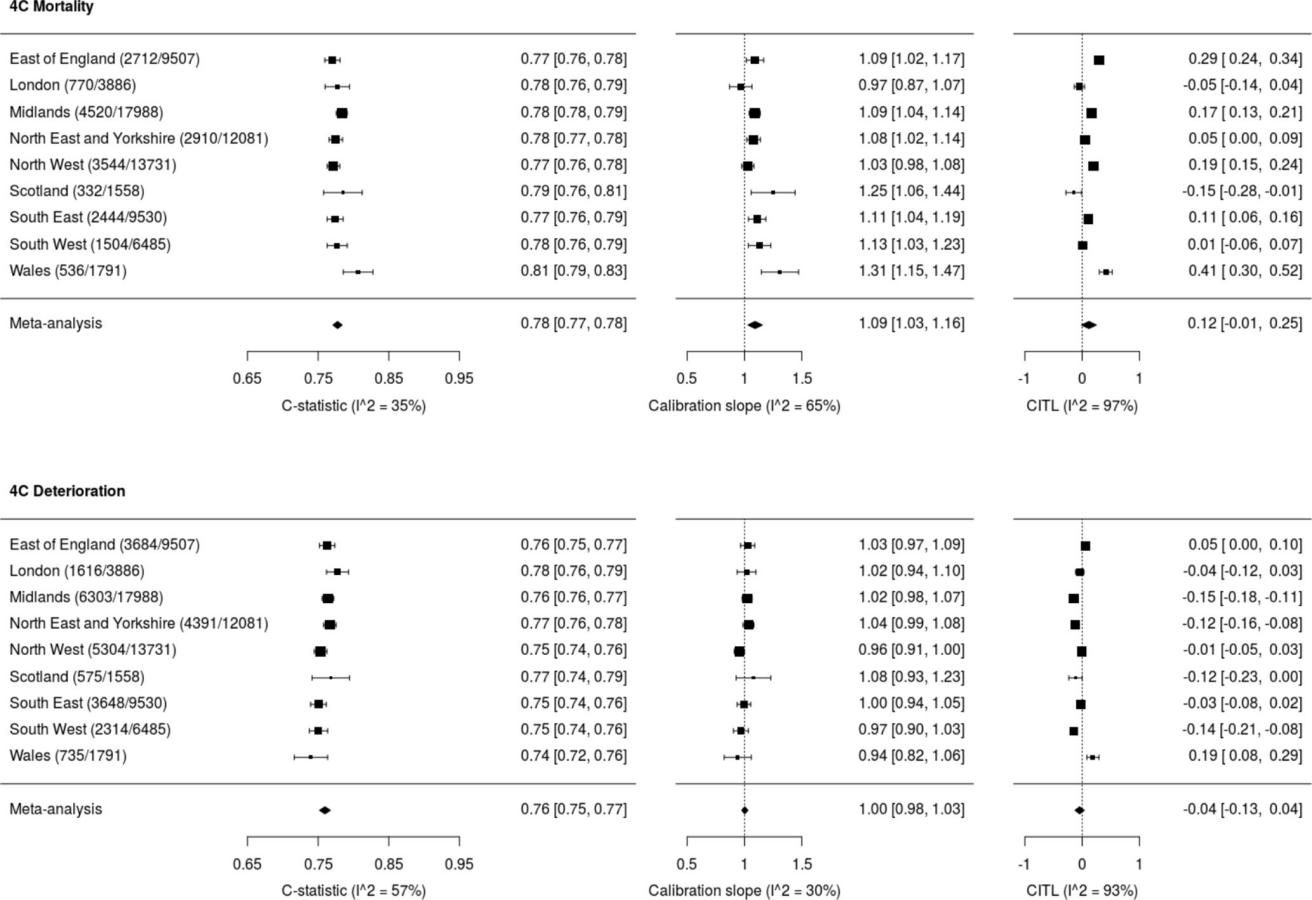
Validation metrics for 4C Mortality and 4C Deterioration score. Random-effects meta-analysis was performed across NHS regions for each metric. CITL, calibration-in-the-large; NHS, National Health Service.

**Figure 3:**
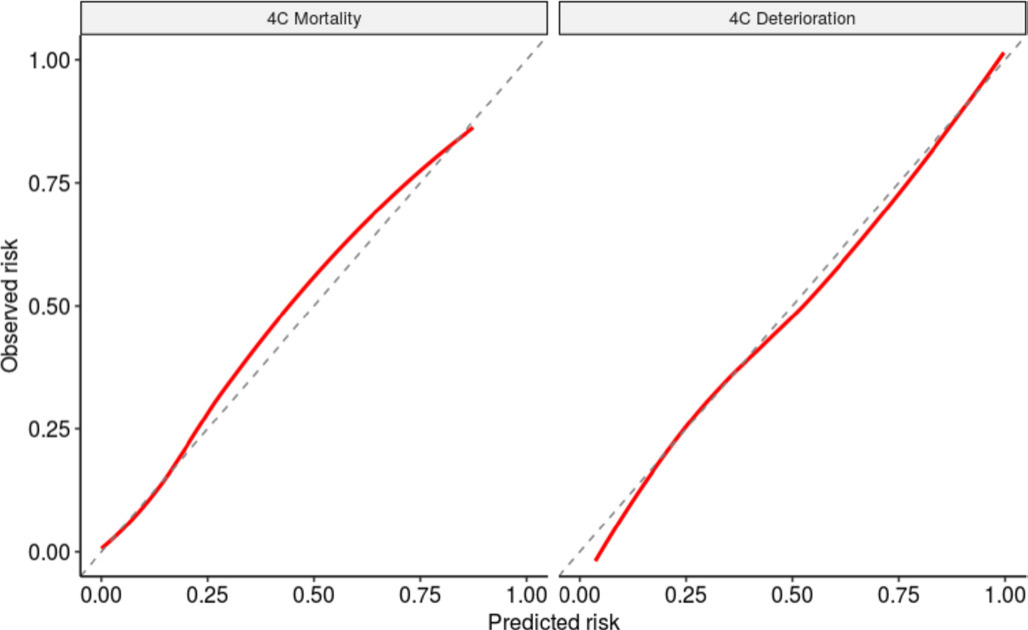
Predicted vs observed outcome probability shows as calibration plots across all NHS regions for 4C Mortality (calibration slope 1.09, CITL 0.12) and 4C Deterioration score (calibration slope 1.00, CITL −0.04). Scores fitted using original derivation cohorts, with predictions from 10 multiply imputed validation data sets, pooled and LOESS curve fitted through predictions. CITL, calibration-in-the-large; NHS, National Health Service.

Forest plots showing model discrimination (C-statistic) and calibration metrics (slope and calibration-in-the-large) for both 4C Mortality and 4C Deterioration scores are shown in figure 2. C-statistics were consistent across NHS regions for 4C Mortality scores (point estimates 0.77–0.81; pooled random-effects meta-analysis estimate 0.78 (95% CI 0.77 to 0.78), I^2^=35%) and 4C Deterioration (point estimates 0.74–0.78; pooled random-effects meta-analysis estimate 0.76 (0.75 to 0.77), I^2^=57%). Calibration slopes were also consistent across regions, for the 4C Mortality score (0.97–1.31; pooled estimate 1.09 (1.03 to 1.16), I^2^=65%) and 4C Deterioration (point estimates 0.94–1.04; pooled estimate 1.00 (0.98 to 1.03), I^2^=30%; figure 3).

Heterogeneity across NHS regions in calibration-in-the-large was seen. For 4C Mortality, points estimates were –0.15 to 0.41 (pooled estimate 0.12 (–0.01 to 0.25), I^2^=97%); for 4C Deterioration, point estimates ranged from –0.15 to 0.19 (pooled estimate –0.04 (–0.13 to 0.04), I^2^=93%). The sensitivity, specificity, positive-predictive value and negative-predictive value across the full range of probability thresholds from the model are shown in online supplemental appendix S3 and S4. Decision curve analysis to further examine clinical utility showed higher net benefit than the treat-all and treat-none approaches across a range of threshold probabilities for both scores (online supplemental appendix S5). Threshold performance and clinical utility were similar to those originally reported for both scores.^2 3^

### Sensitivity analysis

Sensitivity analyses that used complete case data showed similar discrimination and performance metrics (online supplemental appendices S6 and S7) to analyses that used the imputed dataset. After stratification of the validation cohort by ethnicity (online supplemental appendices S8 and S9), discrimination remained similar for the two scores (4C Mortality range 0.77–0.83; 4C Deterioration 0.75–0.82). However, discrimination appeared marginally better among all non-white ethnic groups (4C Mortality range 0.82–0.83; 4C Deterioration 0.79–0.82) compared with the white group (4C Mortality range 0.77 (95% CI 0.76 to 0.77)); 4C Deterioration 0.75 (95% CI 0.75 to 0.76). Calibration-in-the-large for black ethnicity demonstrated some evidence of inconsistency for both 4C Mortality (−0.13 (95% CI −0.30 to 0.04)) and 4C Deterioration (−0.26 (95% CI −0.38 to −0.14)), particularly in complete case analysis (online supplemental appendix S9), despite excellent discrimination (4C Mortality 0.83 (95% CI 0.80 to 0.85)); 4C Deterioration 0.79 (95% CI 0.76 to 0.81).

Despite changes in unadjusted mortality when stratified by month of admission for patients aged ≥60 years old (online supplemental appendix S10), discrimination (4C Mortality range 0.77–0.80, I^2^=74%; 4C Deterioration 0.75–0.79, I^2^=82%) and calibration slopes (4C Mortality point estimates 1.05–1.16, I^2^=6%; 4C Deterioration 0.97–1.09, I^2^=49%, online supplemental appendices 11–13) were found to remain stable for both scores. However, 4C Mortality demonstrated heterogeneity for calibration-in-the-large in December 2020 (0.27) compared with other months of admission (range −0.06 to 0.14, pooled estimate 0.10 (−0.02 to 0.22), I^2^=96%; online supplemental appendix S11), corresponding with an increase in unadjusted mortality for patients ≥70 years old (online supplemental appendix S10). The calibration plot for patients recruited during December demonstrated an associated underestimation of mortality risk (online supplemental appendix S12). For 4C Deterioration, calibration-in-the-large was similar across admission month (pooled estimate −0.06 (−0.11 to −0.01), I^2^=81%).

**Figure 4:**
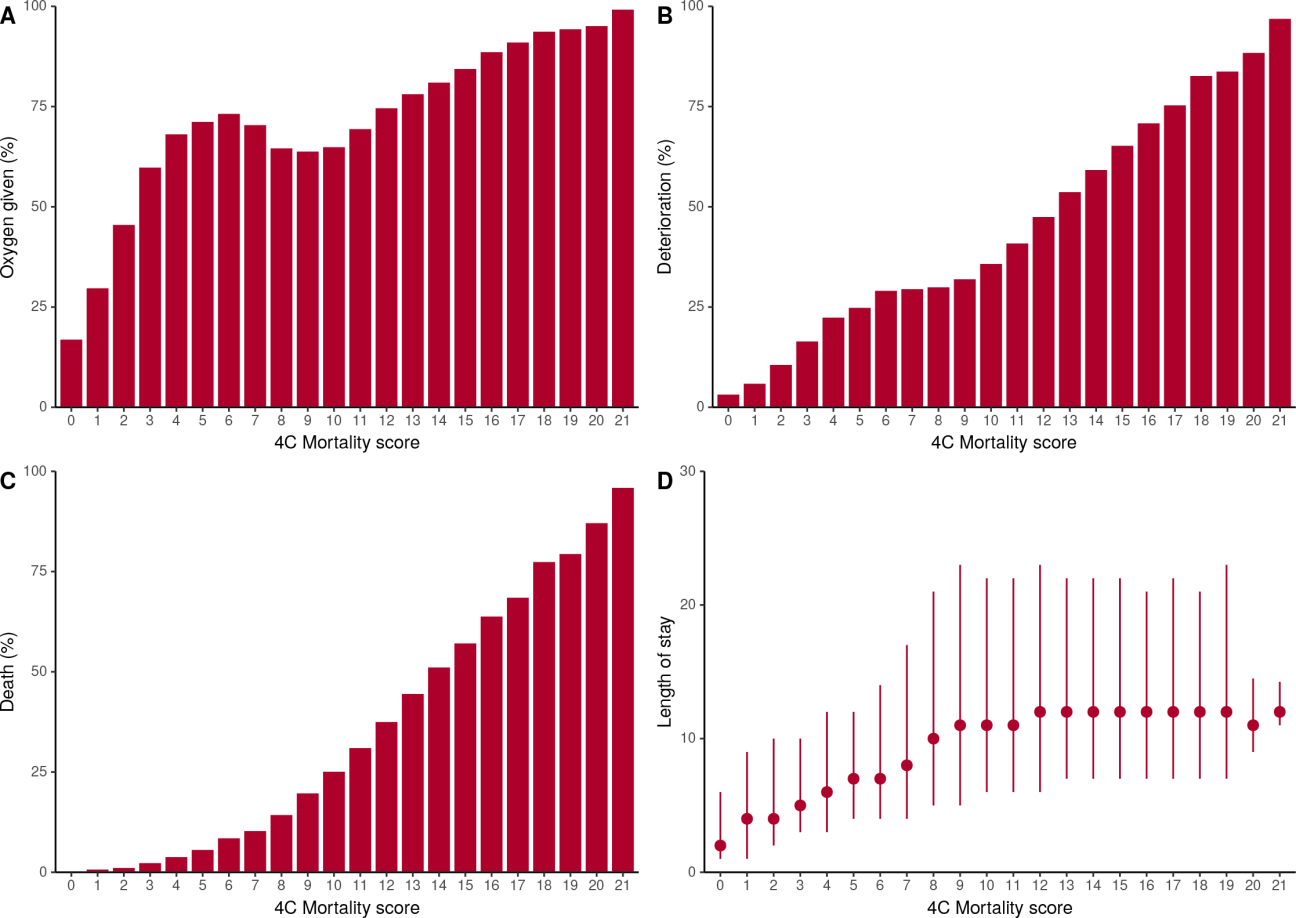
Risk of oxygen requirement, deterioration, death and length of stay stratified 4C Mortality score.

**Figure 5:**
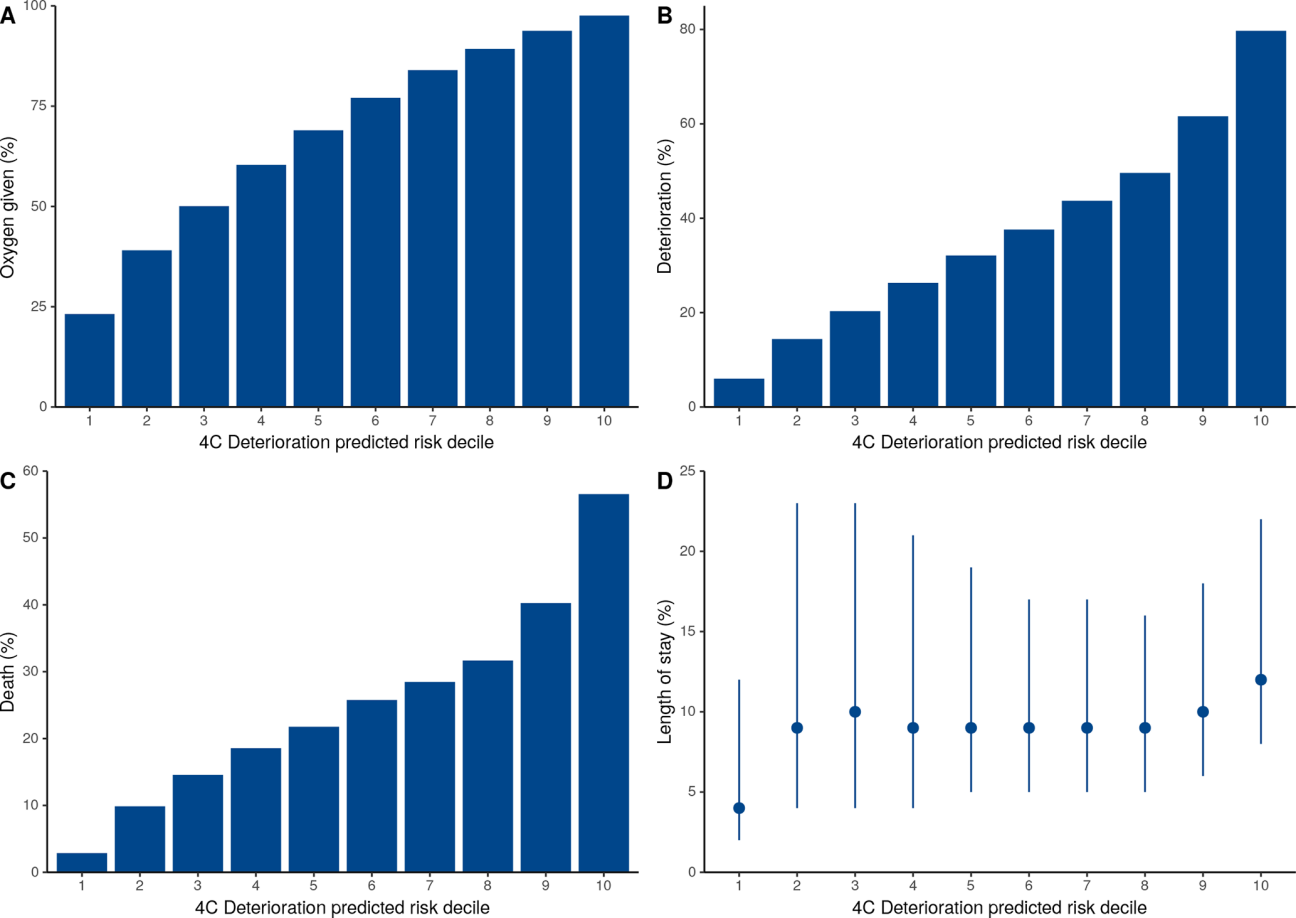
Risk of oxygen requirement, deterioration, death and length of stay stratified by 4C Deterioration score predicted risk deciles.

Across the four 4C Mortality risk groups, the corresponding mortality risks were: low risk (0–3 score, mortality rate 1.5%); intermediate risk (4–8 score, 9.5%); high risk (9–14 score, 32.8%); and very high risk (≥15 score, 63.9%). These mortality risks were similar to the original validation cohort (online supplemental appendices 14 and 15). A stepwise increase in oxygen requirement, deterioration, mortality and duration of hospital stay was seen for both scores as the predicted outcome risk increased (figures 4 and 5). For those identified at the lowest risk of mortality (0–3 score) or deterioration (first and second deciles), patients were the least likely to receive oxygen or deteriorate and had a shorter length of in-patient stay.

## Discussion

### Principal findings

We prospectively validated our previously reported risk stratification tools in a prospective cohort study of 76 588 UK hospitalised patients with confirmed or highly suspected COVID-19 during the second pandemic wave. Both scores (4C Mortality and 4C Deterioration) showed consistent discrimination and calibration across NHS regions and ethnicity. Similar metrics to the original reported first wave validation cohorts were found, despite systematic changes in patient management compared with the first wave, particularly the routine use of corticosteroids.

Robust prognostic models that predict outcomes among COVID-19 patients have been urgently needed to support clinical decision-making regarding hospital admission and treatment. Our 4C scores measure patient comorbidity, abnormal physiology and inflammation using routinely measured demographics, bedside observations and laboratory tests to facilitate objective evidence-based assessments. We previously noted that our prognostic tools should be interpreted in the context of current standard treatment at the time the models were developed and validated.^12^ Hospital-based management of adults with COVID-19 has evolved with the accrual of new evidence and increasing clinical experience, including dexamethasone use and patient proning.^11 27–29^ Therefore, the temporal assessment of model performance is critical to ensure consistent performance and inform the need for temporal recalibration, by updating model intercepts, slopes or coefficients as required.^12^ Our findings suggest that recalibration is currently not required for the 4C Mortality score or 4C Deterioration model. We will continue to monitor model performance prospectively in ISARIC4C and can perform temporal recalibration to update the model if required.

### Clinical management

A key aim of risk stratification is to support clinical management decisions, as part of daily routine care and inform stratification of patients on the basis of clinical severity. The combination of both scores could be included in the programmatic standard of care adopted by hospitals to identify clinical pathways for patients with COVID-19. As well as improving clinical management, it should also encourage better allocation of human and economic resources.^30^

We demonstrate that risk classes identified in original score derivation and validation cohorts continue to perform well, with a stepwise elevation in oxygen requirement, risk of deterioration and death seen as risk class increased. In addition, both scores performed well across the full range of risk. Patients identified as low risk by both scores were less likely to require oxygen and have a short hospital admission, suggesting these patients could be managed in the community if supported by a clinician’s overall assessment, while patients within the intermediate-risk group (4C Mortality) or third to fifth deciles (4C Deterioration) might be suitable for ward level monitoring initially. Meanwhile, patients at high risk of mortality or deterioration may prompt aggressive treatment and early escalation to critical care if appropriate. However, these risk stratification tools should guide, but not replace, clinical decision making. Furthermore, care should be taken when interpreting predicted risk, as it does not reflect the outcome risk in the absence or presence of a particular intervention.^30^ Unfortunately, the prediction of risk with and without intervention is difficult to perform.^31^

### Comparison with other studies

Consistent performance of both models is perhaps surprising, particularly as the introduction of corticosteroids is likely to have reduced mortality among people receiving supplementary oxygen.^11 32^ In this cohort, 38 417 (54%) patients received systemic corticosteroids compared with 14% and 12% in the original 4C Deterioration and Mortality cohorts. On the contrary, the B.1.1.7 variant was reported to be the dominant circulating SARS-CoV-2 strain during the second wave^33^ and may be associated with higher mortality.^34^ In our present study, mortality (17%) and deterioration (37%) rates were lower than cohorts used initially for model derivation and validation (mortality 32%, deterioration 43%) despite similar patient age, comorbidities and time to outcome. Nonetheless, both models performed well despite these changes across the included time period. Therefore, application of the 4C Mortality and 4C Deterioration scores together still provides a validated and evidence-based approach for clinicians to predict the appropriate outcome as required to inform clinical management decisions.

### Strengths and limitations of this study

This is the largest prospective validation study for prognostic risk scores among hospitalised patients with COVID-19. As for our original reports, we adhered to TRIPOD reporting standards,^18^ used multiple imputation to deal with missing data and examined heterogeneity in detail by NHS region, ethnicity and month of admission. We have demonstrated the ability to temporally validate both scores during subsequent admission waves and, if required, can temporally recalibrate both scores if performance decreases in future. In addition, both scores were able to identify both low-risk (rule-out) and high-risk patients (rule-in) for mortality and deterioration, which corresponded with oxygen requirement and duration of in-hospital stay.

There are, however, some limitations. First, the patient cohort comprised of hospitalised patients with confirmed or highly suspected COVID-19 who were seriously ill (mortality rate of 17.4%) and were of advanced age (median age 72 years), similar to cohorts used to derive each score. These models are not for use in the community and could still perform differently in populations at lower risk of death. In addition, pooled estimates demonstrated high heterogeneity, particularly for calibration-in-the-large. This may represent clinical differences across each region, including patient population and the medical management of hospitalised patients with COVID-19. Nevertheless, external validation in Brazil,^6^ Canada,^7^ France,^8^ Netherlands^9^ and Pakistan^10^ has demonstrated consistent performance for the 4C Mortality Score.

Second, a proportion of recruited patients had incomplete episodes for deterioration (4.5%) or mortality (5.4%). We handled missing outcome data using multiple imputation in the primary analysis, assuming missingness at random and performed a complete case sensitivity analysis, with consistent findings. Furthermore, the inclusion of all-cause mortality as a primary outcome measure, rather than covid-related mortality, may reduce interpretability in patients with nosocomial infection.

## Conclusions and policy implications

We have prospectively validated easy-to-use risk scores that enable accurate stratification among hospitalised adults with community-acquired or hospital-acquired COVID-19 for clinical deterioration or mortality. The performance of both scores has remained consistent despite temporal changes in management and treatment during the second wave. Application within the validation cohorts showed this tool could guide clinician decisions, including treatment escalation. Although the models showed consistent performance across England, Wales and Scotland, validation in other countries should be prioritised to enable its clinical implementation internationally. The ongoing performance of both scores will need to be assessed in the context of increasing deployment of immunomodulatory agents^35^ and COVID-19 vaccines, as well as emerging SARS-CoV-2 variants.

## Data Availability

Access to all data and samples collected by ISARIC4C are controlled by an Independent Data and Materials Access Committee composed of representatives of research funders, academia, clinical medicine, public health, and industry. The application process for access to the data is available on the ISARIC4C website (https://isaric4c.net/sample_access/).
