# Supplementary material for: Prospective validation of the 4C prognostic models for adults hospitalised with COVID-19 using the ISARIC WHO Clinical Characterisation Protocol
Source: Thorax. 2021 Nov 22;77(6):606–15. doi: 10.1136/thoraxjnl-2021-217629 (PMC8610617; doi:10.1136/thoraxjnl-2021-217629)
Supplement: Supplementary data [file thoraxjnl-2021-217629supp001.pdf]

Supplemental Appendix

S1. Transformation of coefficients to points system for both risk stratification scores

4C Mortality

A three-stage model building process was used. Firstly, generalised additive models were built incorporating continuous smoothed predictors (penalised thin plate splines) in combination with categorical predictors as linear components. A criterion-based approach to variable selection was taken based on the deviance explained, the unbiased risk estimator, and the area under the receiver operating characteristic curve. Secondly, we visually inspected plots of component smoothed continuous predictors for linearity, and selected optimal cut-off values by using the methods of Barrio and colleagues (doi: 10.1186/1471-2288-13-83). Lastly, the final models using categorised variables were specified with least absolute shrinkage and selection operator logistic regression. L1 penalised coefficients were derived using 10-fold cross validation to select the value of lambda (minimised cross validated sum of squared residuals). We converted shrunk coefficients to a prognostic index with appropriate scaling to create the pragmatic 4C Mortality Score.

4C Deterioration

4C Deterioration is a logistic regression model, providing a probability output. To facilitate manual calculation of predicted risk, we have also provided points score transformation at https://isaric4c.net/risk/. To do this, we used the ‘nomogram’ function in the rms package in R (hbiostat.org/doc/rms/4day.html) to allocate a points score for each distinct value of each predictor in the original development cohort. To calculate the 4C Deterioration score for any given patient under evaluation, score allocations for the value of each predictor corresponding to that patient are then summed across all predictors to derive the 'total points score'. The points score allocations for each predictor and the corresponding deterioration 'probability' allocated to that 'total points score' can be found in our online look-up tables (https://isaric4c.net/risk/).

S2. Distribution of missing data across cohort

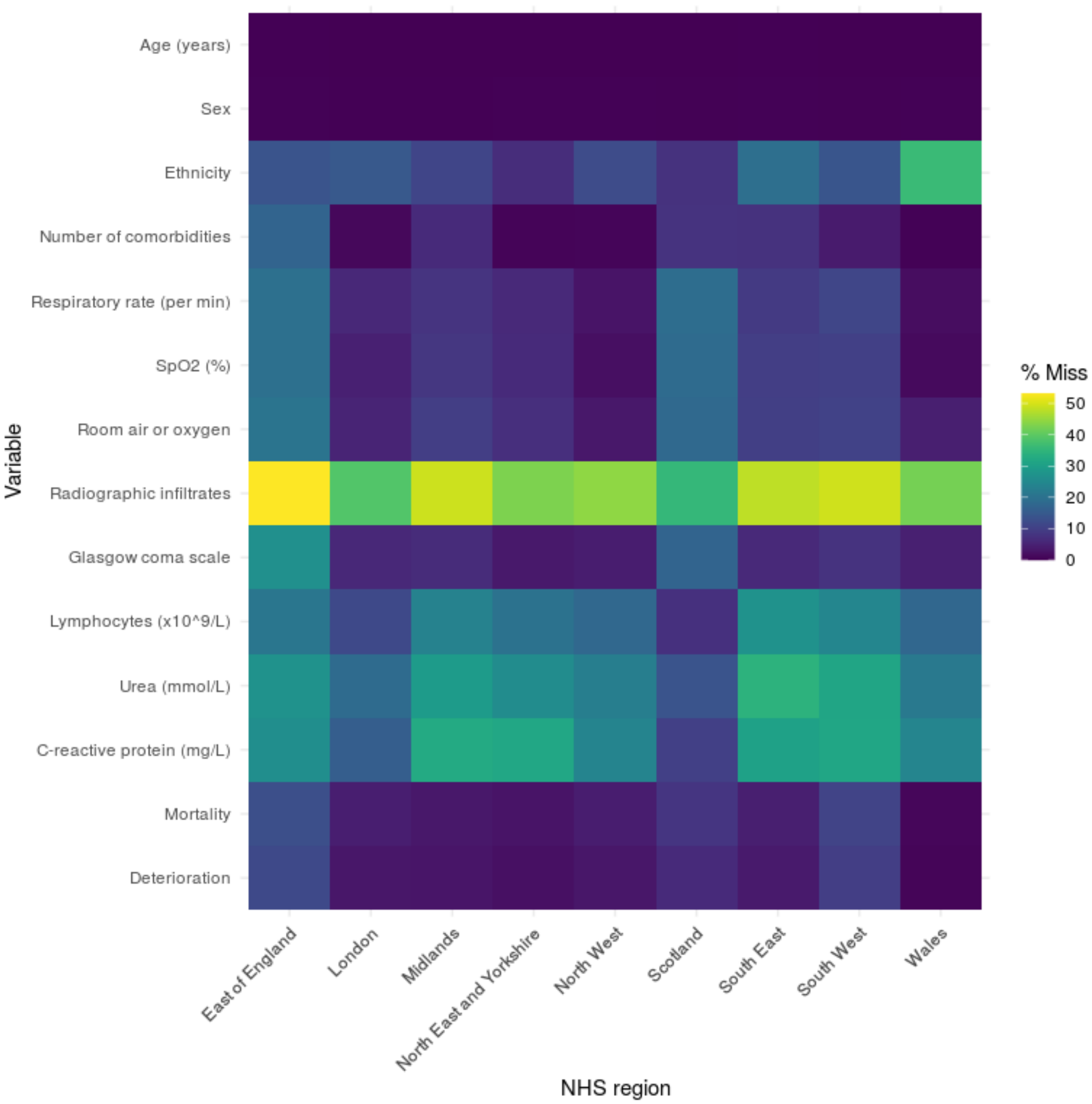

S3. Sensitivity, specificity, positive predictive values (PPVs), and negative predictive values (NPVs) of the 4C Mortality Score within the validation cohort (n=76 588 participants), estimated across each of ten multiply imputed datasets and pooled using Rubin's rules.

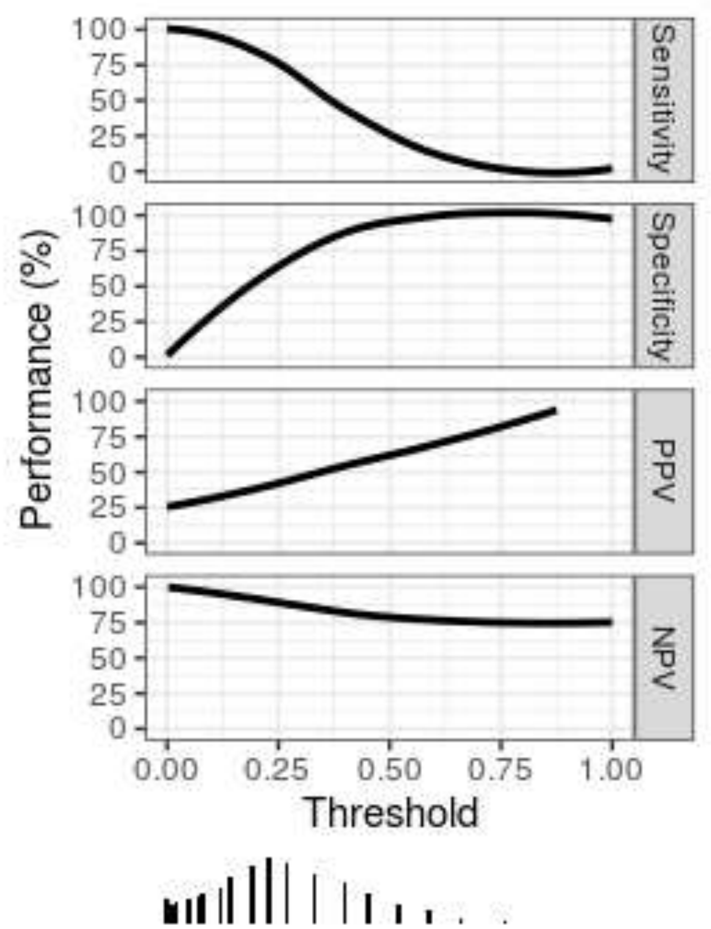

Each metric is shown across the full range of probability thresholds for the model predictions for illustration. The distribution of predictions is shown in the histogram. Positive predictive value at a threshold of 0% and negative predictive values at a threshold of 100% reflect prior probability due to the overall observed risk of mortality in the validation cohort.

S4. Sensitivity, specificity, positive predictive values (PPVs), and negative predictive values (NPVs) of the 4C Deterioration Score within the validation cohort (n=76 588 participants), estimated across each of ten multiply imputed datasets and pooled using Rubin's rules.

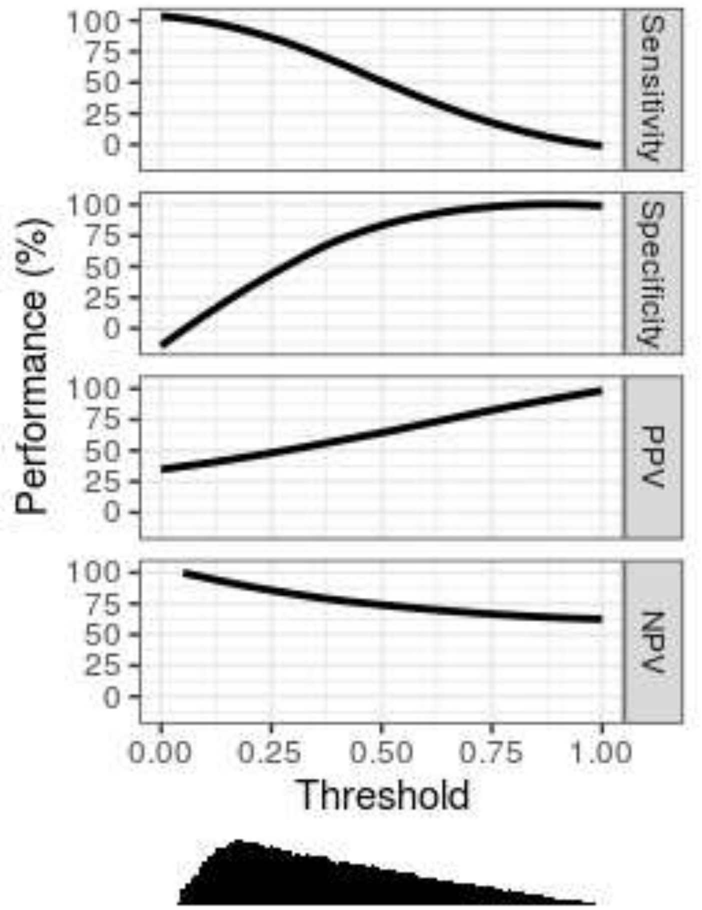

Each metric is shown across the full range of probability thresholds for the model predictions for illustration. The distribution of predictions is shown in the histogram. Positive predictive value at a threshold of 0% and negative predictive values at a threshold of 100% reflect prior probability due to the overall observed risk of deterioration in the validation cohort.

S5. Standardised net benefit at different risk thresholds of treating no patients (black line) and treating all patients (black dashed line) compared to 4C Mortality (A) and 4C Deterioration (B) scores

A

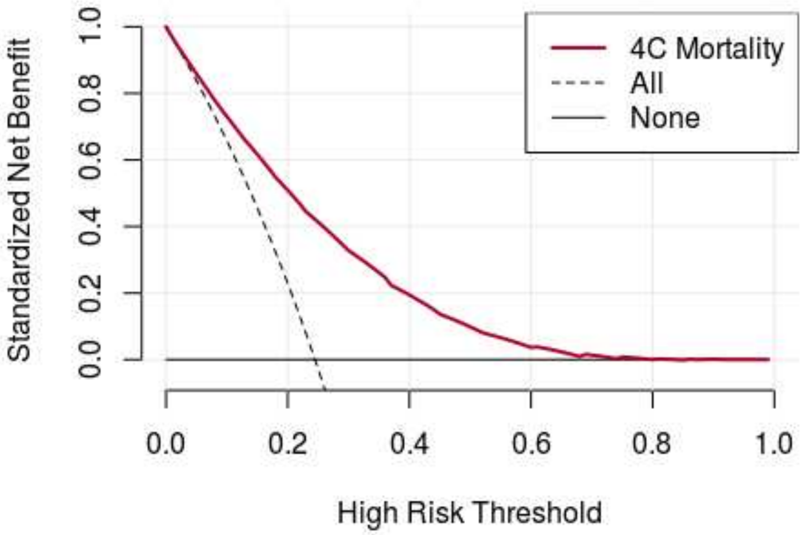

B

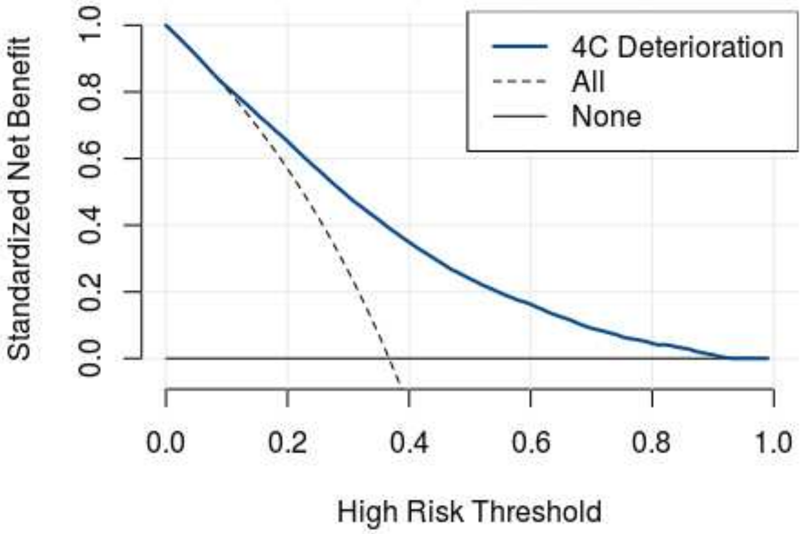

S6. Complete case discrimination and calibration for 4C Mortality and 4C Deterioration Score. Random-effects meta-analysis was performed across each NHS region

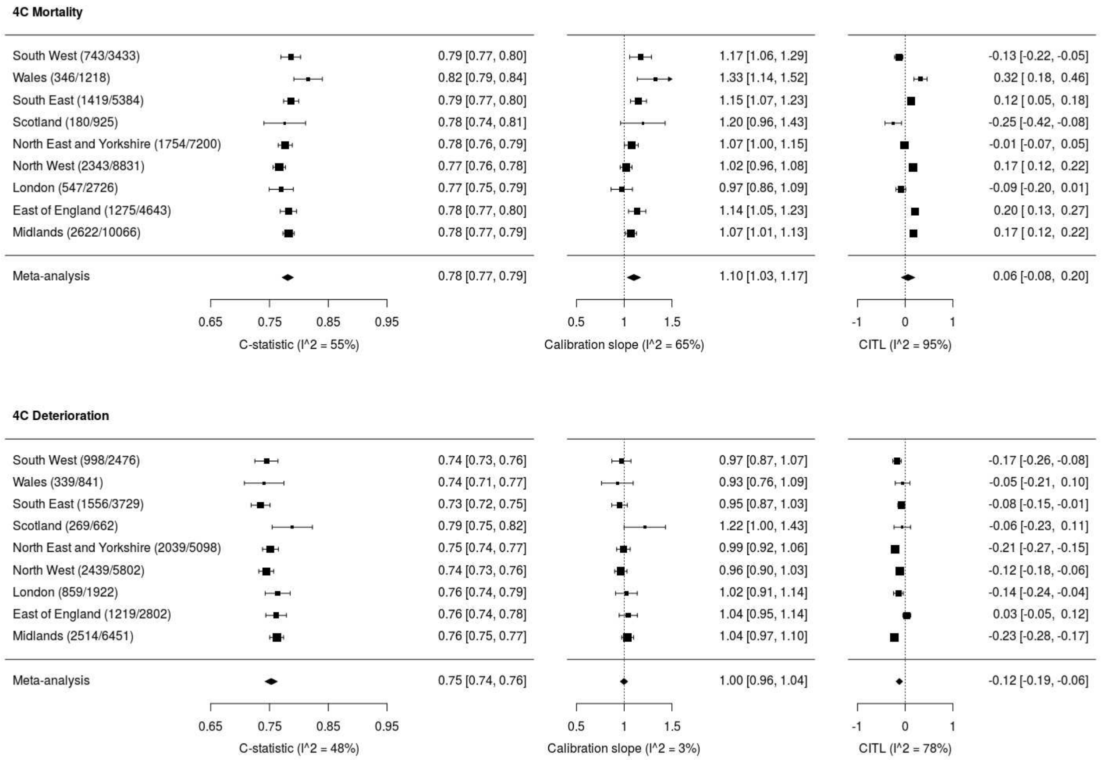

CITL = calibration-in-the-large. Events / N are shown for each region

S7. Calibration curves for 4C Mortality and 4C Deterioration Score across complete case cohort. Scores fitted using original derivation cohorts, with predictions from validation dataset and loess curve fitted through predictions. Grey shading represents 95% confidence intervals

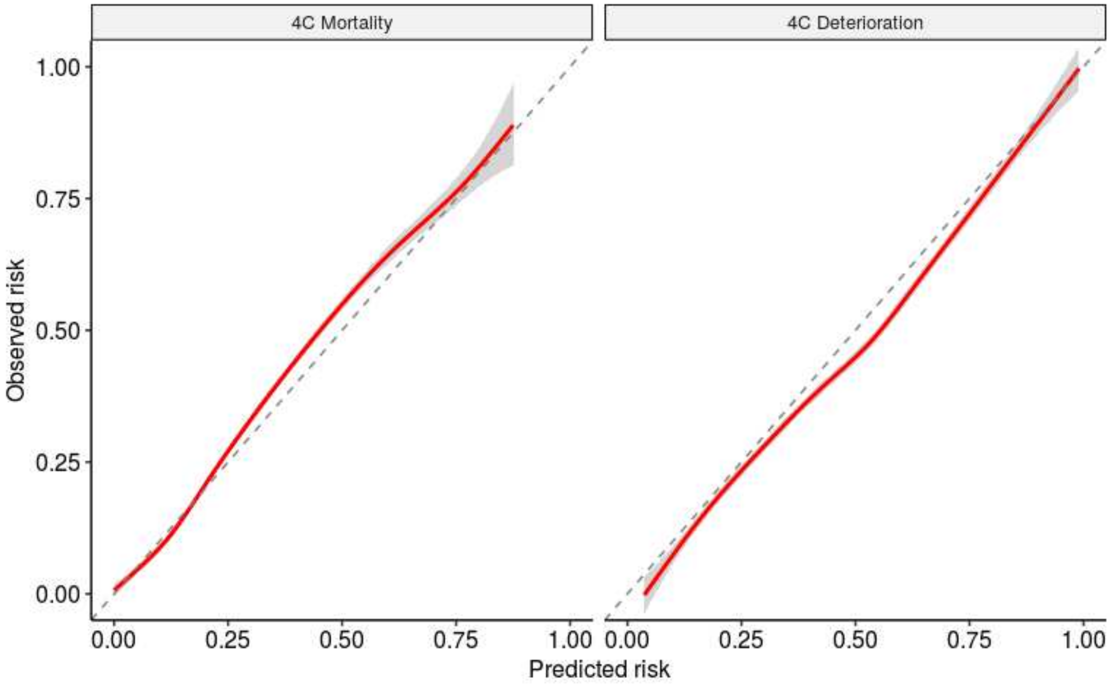

S8. Discrimination and calibration for 4C Mortality and 4C Deterioration Score. Random-effects meta-analysis was performed across ethnicity for each metric

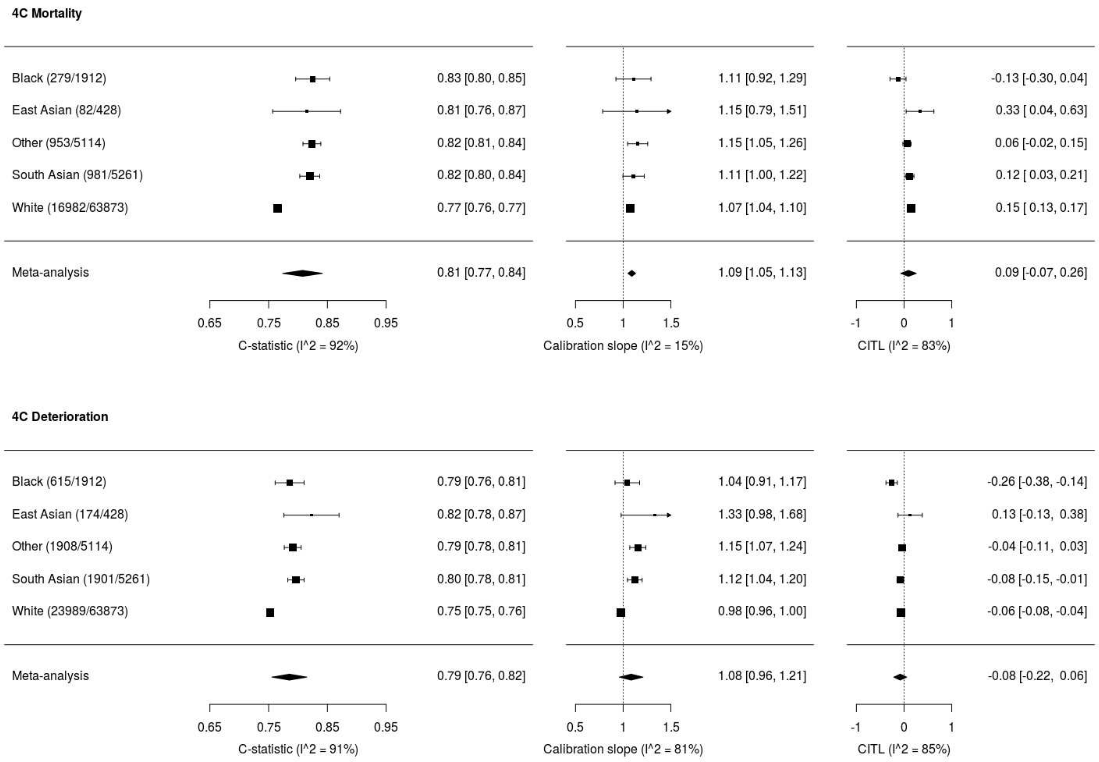

CITL = calibration-in-the-large. Events / N are shown for each region

S9. Complete case discrimination and calibration for 4C Mortality and 4C Deterioration Score. Random-effects meta-analysis was performed across each ethnicity

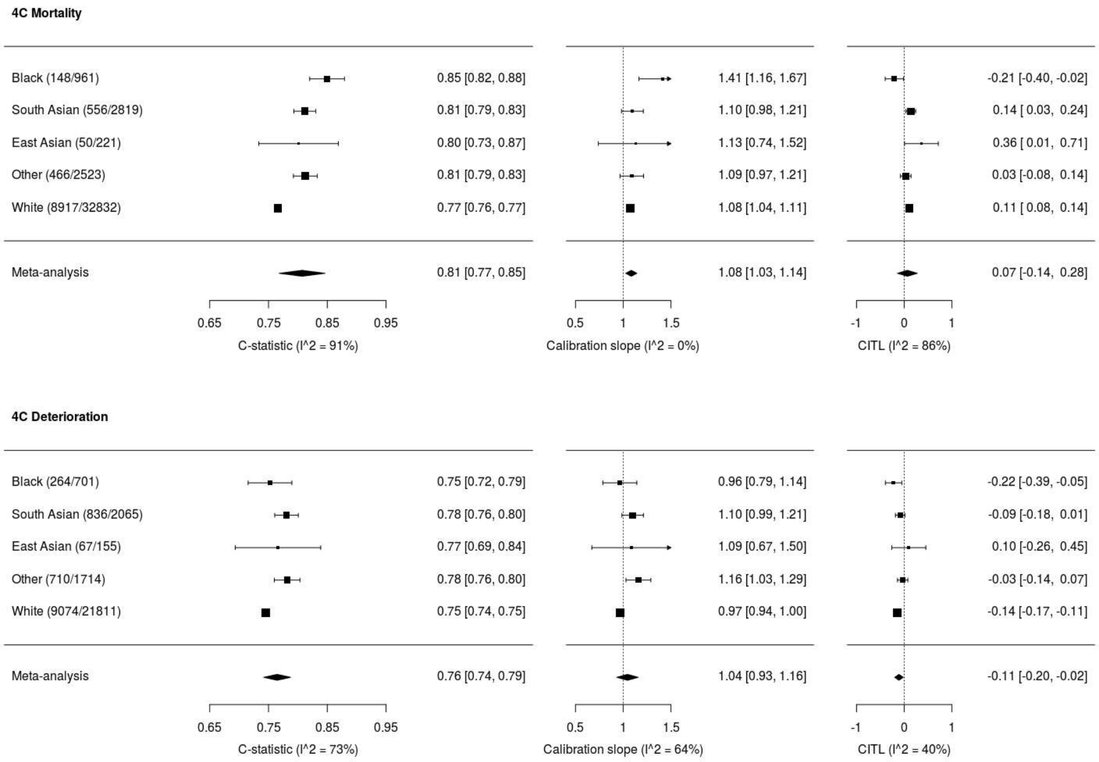

CITL = calibration-in-the-large. Events / N are shown for each region

S10. Change in unadjusted mortality rates over time stratified by age, with loess curve fitted to demonstrate relationship between month of admission and mortality

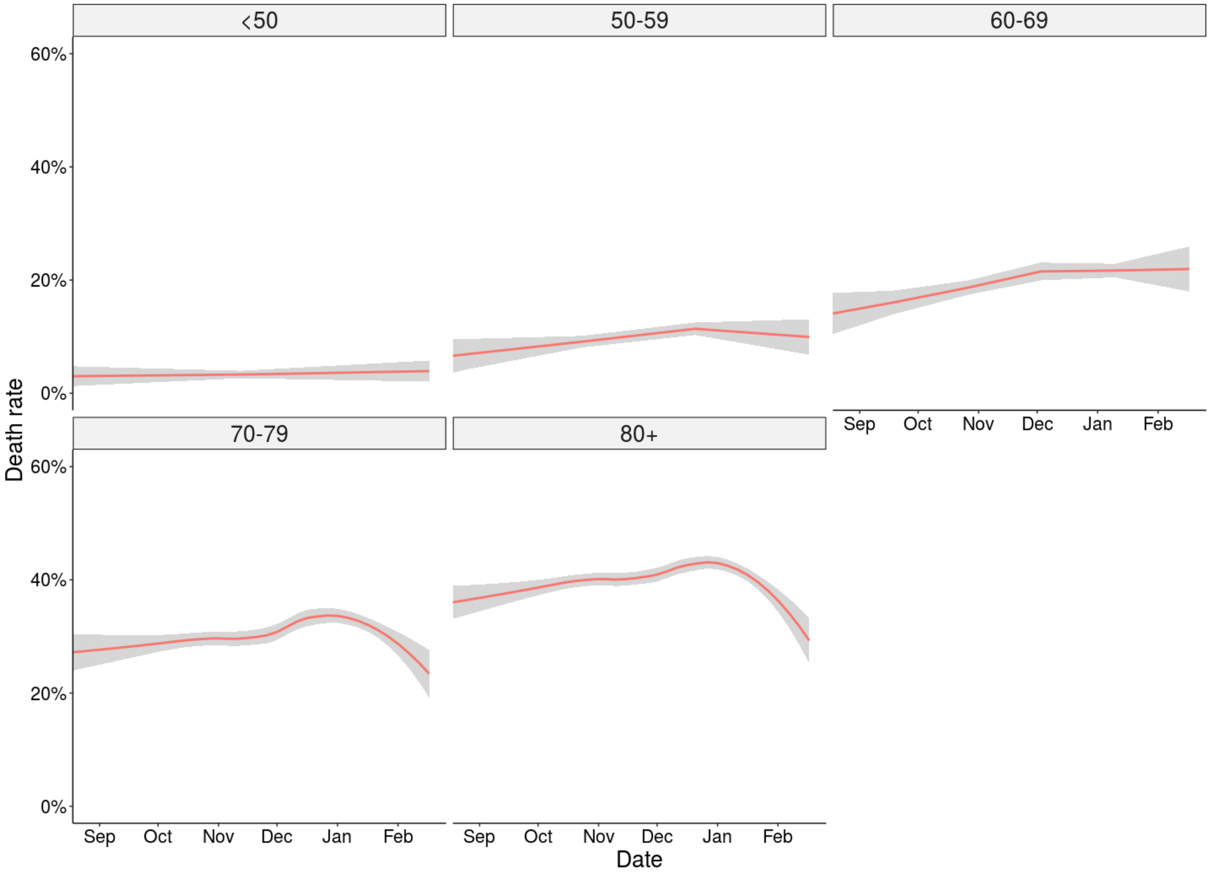

S11. Discrimination and calibration for 4C Mortality and 4C Deterioration Score. Random-effects meta-analysis was performed across month of admission for each metric

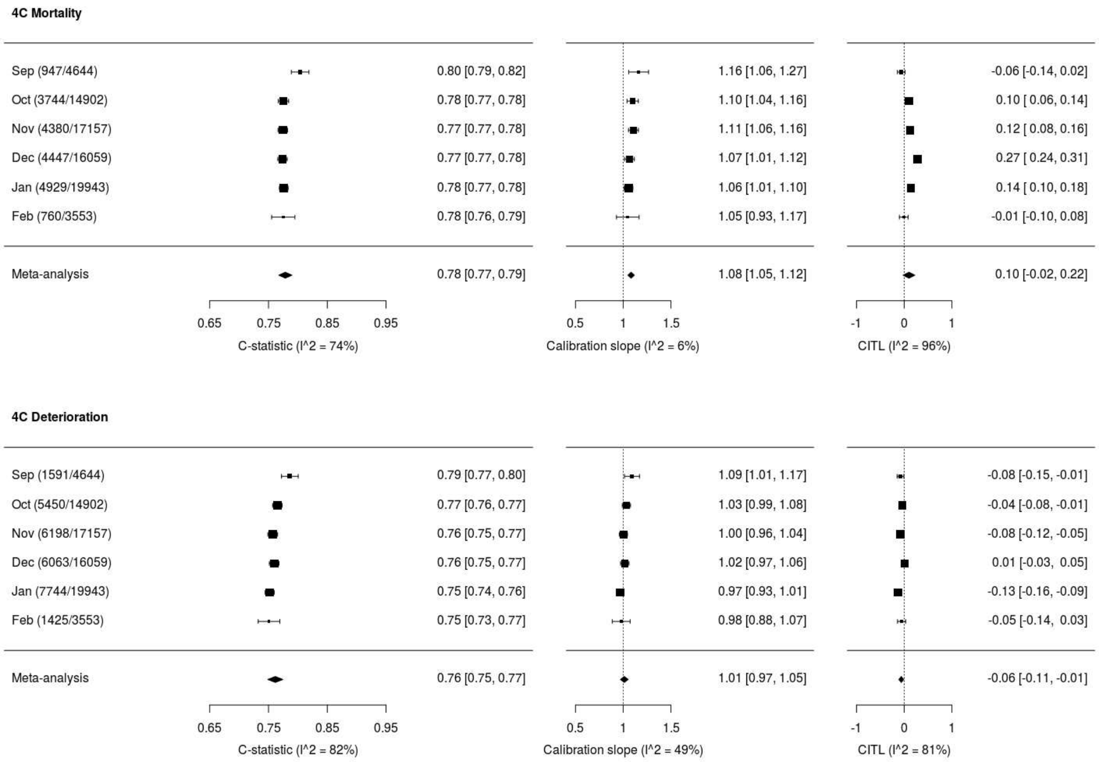

CITL = calibration-in-the-large. Events / N are shown for each region

S12. Predicted versus observed probability of in-hospital mortality (calibration; red line) for 4C Mortality Score by month of admission. Scores fitted using original derivation cohort, with predictions from ten multiply imputed validation data sets, pooled, and loess curve fitted through predictions

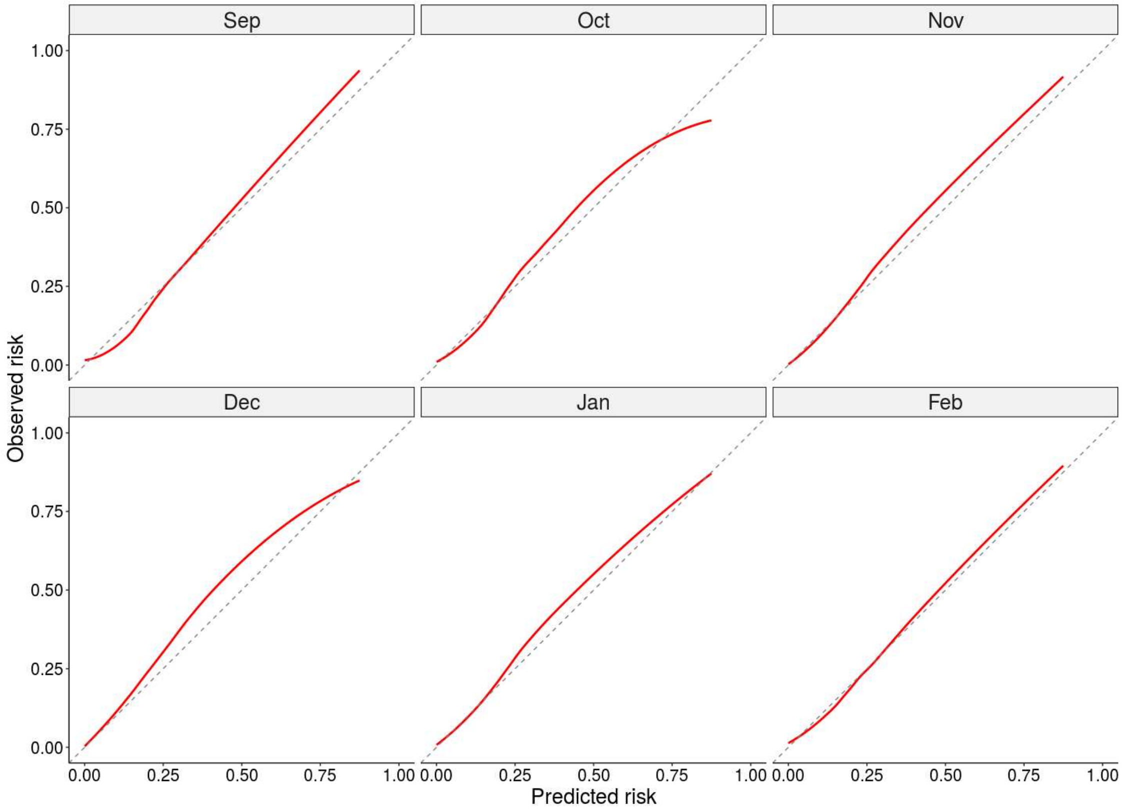

Confidence intervals are not shown as calibration plots were fitted across 10 stacked multiply imputed datasets

S13. Predicted versus observed probability of in-hospital mortality (calibration; red line) for 4C Deterioration Score by admission month. Scores fitted using original derivation cohort, with predictions from ten multiply imputed validation data sets, pooled, and loess curve fitted through predictions

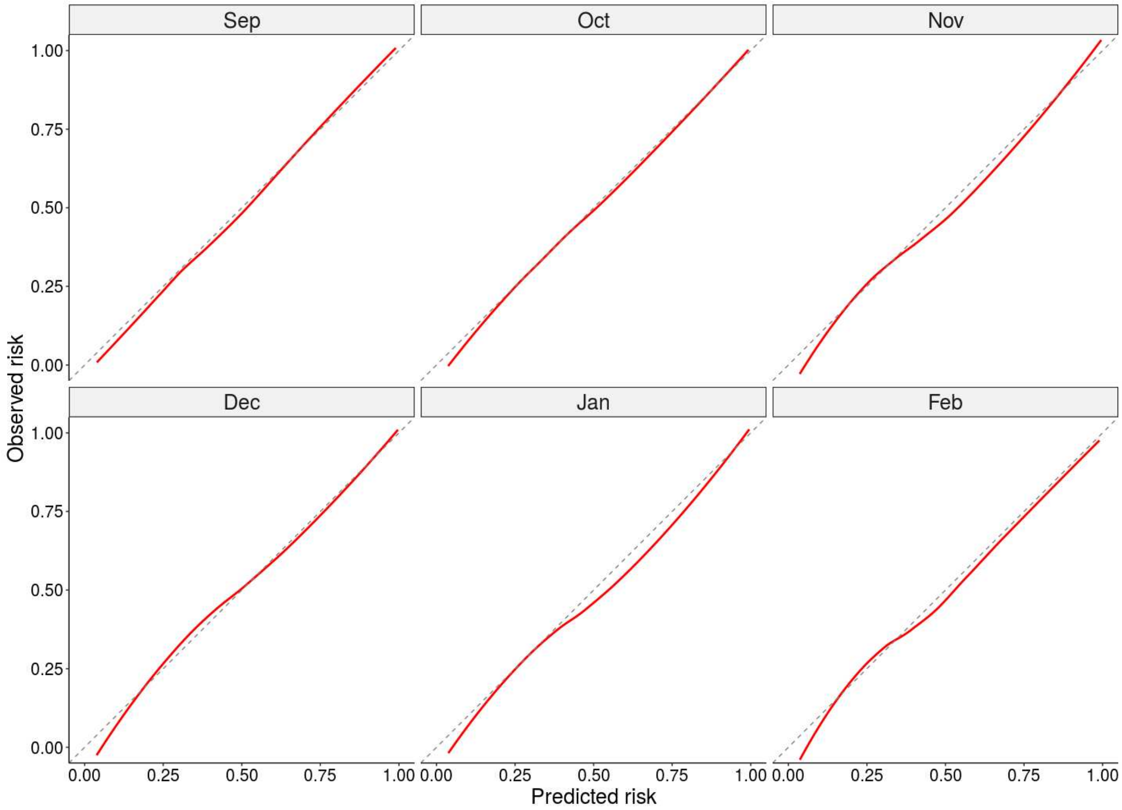

Confidence intervals are not shown as calibration plots were fitted across 10 stacked multiply imputed datasets

S14. Recalibrated 4C Mortality Score probabilities for each index value

4C Mortality Score	4C Mortality Probability (%)	Original validation cohort mortality (%)
0	0.4	0.0
1	0.9	0.3
2	1.3	0.8
3	2.5	2.3
4	4.0	4.8
5	5.8	7.5
6	8.7	7.8
7	10.5	11.7
8	14.5	14.4
9	19.9	19.2
10	25.3	22.9
11	31.2	26.9
12	37.7	32.9
13	44.7	40.1
14	51.3	44.6
15	57.3	51.6
16	64.0	59.1
17	68.7	66.1
18	77.6	75.8
19	79.6	77.3
20	87.3	82.9
21	96.1	87.5

S15. Comparison of mortality rates for 4C Mortality Score risk groups across original and updated validation cohorts

	Validation cohort		Original validation cohort	
Risk group	Number of patients (%)	Mortality (%)	Number of patients (%)	Mortality (%)
Low (0-3)	8631 (11.3)	1.5	1650 (7.4)	1.2
Intermediate (4-8)	21682 (28.3)	9.5	4889 (21.9)	9.9
High (9-14)	40106 (52.4)	32.8	11 664 (52.1)	31.4
Very high (>14)	6169 (8.0)	63.9	4158 (18.6)	61.5
